# Limited flexibility in departure timing of migratory passerines at the East-Mediterranean flyway

**DOI:** 10.1038/s41598-021-83793-x

**Published:** 2021-03-04

**Authors:** Yaara Aharon-Rotman, Gidon Perlman, Yosef Kiat, Tal Raz, Amir Balaban, Takuya Iwamura

**Affiliations:** 1 School of Zoology, Faculty of Life Sciences, Tel Aviv University, 6997801 Tel Aviv, Israel; 2 School of Environmental and Rural Science, University of New England, Armidale, NSW 2351 Australia; 3 The Nili and David Jerusalem Bird Observatory, Society for the Protection of Nature in Israel, Jerusalem, Israel; 4 Department of Evolutionary and Environmental Biology and the Institute of Evolution, University of Haifa, 3498838 Haifa, Israel; 5 Department of Forest Ecosystems and Society, College of Forestry, Oregon State University, Corvallis, OR USA

**Keywords:** Ecology, Zoology, Animal behaviour

## Abstract

The rapid pace of current global warming lead to the advancement of spring migration in the majority of long-distance migratory bird species. While data on arrival timing to breeding grounds in Europe is plentiful, information from the African departure sites are scarce. Here we analysed changes in arrival timing at a stopover site in Israel and any links to Enhanced Vegetation Index (EVI) on the species-specific African non-breeding range in three migratory passerines between 2000–2017. Differences in wing length between early and late arriving individuals were also examined as a proxy for migration distance. We found that male redstart, but not females, advanced arrival to stopover site, but interestingly, not as a response to EVI phenology. Blackcap and barred warbler did not shift arrival timing significantly, although the arrival of blackcap was dependent on EVI. Barred warbler from the early arrival phase had longer wings, suggesting different populations. Our study further supports the existence species-specific migration decisions and inter-sexual differences, which may be triggered by both exogenous (local vegetation condition) and endogenous cues. Given rapid rate of changes in environmental conditions at higher latitudes, some migrants may experience difficulty in the race to match global changes to ensure their survival.

## Introduction

Current global warming is occurring at an asymmetrical pace, with greater increase in temperatures at higher northern latitudes^1,2^. Migratory birds are particularly sensitive to climatic changes, as they depend on multiple sites with spatial variability in conditions along their migration route. Many migrants show some degree of adjustment in response to global changes by changing migration speed, phenology or stopover duration e.g.^3–5^, while others do not exhibit such responses e.g.^6,7^. Failing to adjust to environmental changes at a sufficient pace, e.g. mismatch between arrival timing and optimal condition of food abundance and temperatures, may therefore have detrimental consequences for bird populations^8–10^. The ability to adjust to changing climates, however, varies among species^11–13^ and may be related to migration distance, environmental conditions or body size^4,7,13–16^.

The decision when to depart from the non-breeding site on spring migration is likely dominated by spatial and temporal local resource availability to accumulate sufficient fat stores during the pre-migratory period^16–18^, and may result in either delayed or advanced arrival at the stopover sites^19^. In addition, migration decisions are triggered by endogenous cues such as circannual rhythms and changes in photoperiod^20–22^ and possibly related to hormone secretion, as has been shown in wild garden warblers (*Sylvia borin*) departing from stopover site in Italy^23^. Endogenous cues have been suggested to be especially relevant in migrants that spend the non-breeding season close to the equator, where environmental cues are weak due to the relatively constant conditions^24,25^. It is therefore likely that under constant environmental conditions, the timing of departure is primarily dominated by endogenous cues, while variation in optimal conditions during the pre-migratory fattening period may trigger a change to migration timing. Still, spring migration phenology of most long-distance migrants shows a general advancement trend in recent decades e.g.^3,4,13,26–28^. An evolutionary response to selection for earlier breeders departing from sub-Sahara Africa may explain this trend^3,21^. However, many studies also found correlations between the advancement of spring migration phenology and climatic variables at either non-breeding or stopover sites^3,5,16^.

Current data on phenological changes in migratory passerines in the Eastern-Mediterranean flyway is largely based on data collected at the European breeding grounds, where many migrants advanced their arrival e.g.^29–31^. Although in many species departure timing from the non-breeding ground is a strong predictor of arrival timing at the breeding ground e.g.^32,33^, some long-distance migrants may adjust migration timing along the flyway in response to changing conditions^5,34,35^. The main non-breeding grounds for many long-distance species in this flyway are the Afrotropical zone. The habitat conditions in these areas are considered imperative for their fitness and survival, and extreme climatic events at these sites have been previously correlated with declines in European breeding populations^36–39^. Despite their presumed importance, we still lack critical information on the non-breeding species-specific ranges^40,41^, and only few studies examined the species-specific response to changing conditions at these sites^16,34,42^. Some studies used large-scale climatic indices, such as the North Atlantic Oscillation^43^, but these methods have their obvious accuracy limitations^44^. More studies are therefore needed in the East Mediterranean flyway, where to date there have been no studies looking at these phenological changes together with their possible causes.

To analyse phenological changes in migratory species, we must be aware that the observed migrants comprise individuals from multiple populations, possibly arriving from different geographical locations with different phenological responses^7,45,46^. Wing length measurements could serve as a surrogate parameter to differentiate among spatially-segregated populations in migratory passerines^47,48^. This is based on the correlation between migration distance and wing length^49–52^. Pérez-Tris, et al.^53^, for example, found that the wing length of the blackcap increased from sedentary to migrant populations, but that this increase levelled-off if migration distance was over 2000 km. Only a handful of studies have examined long-term changes in wing length^54,55^ and related it to climatic changes^56^, indicating local morphological adaptations to distinct migratory routes and over a relatively small spatial scale^52^.

The aim of our study is to estimate changes in spring arrival phenology and the effect of environmental factors at the Afrotropical non-breeding grounds on the arrival timing of three long-distance migratory passerine species at a stopover site in Israel between 2000–2017. To this end, we used a combination of long-term observations with satellite-based spatial environmental information. We also compare the wing length of early and late arriving individuals, to discern between migration distances (i.e. populations) and analyse long-term changes in wing length as a possible indication for phenotypic adaptation. We predict that increased vegetation index will facilitate early departure (and arrival to stopover site) due to changes of resources in the critical pre-migratory fattening period, and that birds departing from sites with little inter-annual environmental variation will have a relatively constant arrival timing, likely because endogenous cues will primarily dominate the decision when to depart.

## Materials and methods

### Study area

The Nili and David Jerusalem Bird Observatory (JBO) in Israel (31.780° N, 35.206° E) is an important stopover site for species that migrate between the Afrotropical non-breeding grounds and breeding grounds in the Palearctic region. The JBO is located in the city of Jerusalem within a large area of parks surrounding the Israeli Parliament building. The elevation is 805 m above sea level and the climate is Mediterranean, with an average annual rainfall of approximately 550 mm. Low trees and bushes typical of the Judean mountains dominate the vegetation. Typical plants are *Pistacia palaestina*, *Rhamnus alaternus*, *Prunus dulcis* (almond), and *Olea europea* (olive). At the center of the site is a small pond surrounded by *Typha domingensis*.

### Species and data collection

**Figure 1:**
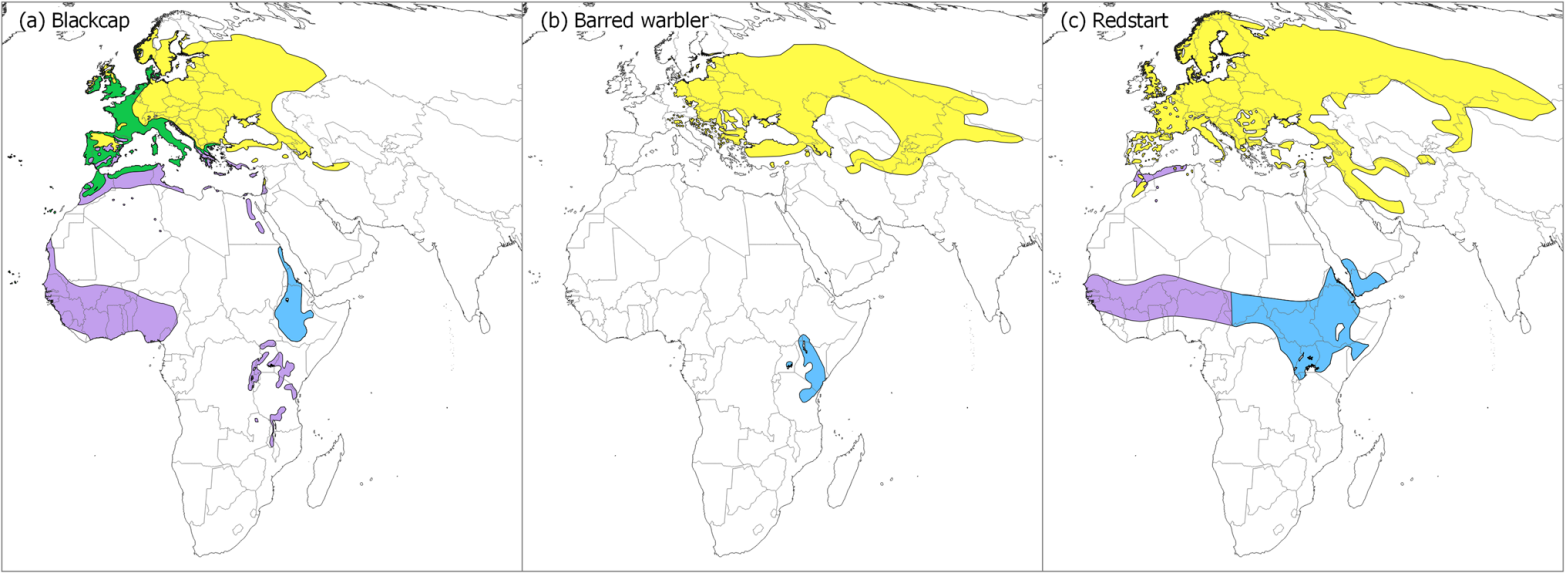
Distribution maps of the study species along the Eastern-Mediterranean flyway. (**a**) Eurasian blackcap (*Sylvia atricapilla)*, (**b**) Barred warbler (*Sylvia nisoria*) and (**c**) Common redstart (*Phoenicurus Phoenicurus*). Yellow depicts breeding ranges; green depicts areas were population stay the entire year; blue depicts presumed non-breeding eastern distribution ranges, where migrants that stopover in Israel are likely to spend the non-breeding season, and purple depicts the western non-breeding distribution. Maps were created using qgis 3.6.0 (qgis 2018, http://qgis.osgeo.org), based on GIS data downloaded from BirdLife International data zone (http://www.birdlife.org/). The non-breeding areas (blue) that were used to calculate Enhanced Vegetation Index (EVI) for the species-specific non-breeding range were selected using qgis, based on the parameter “season” of the attribute table of the dataset.

Many species present sexual morphological and phenological differences^57^, with males arriving earlier at the site e.g.^58–60^. We therefore chose to analyse only species for which the sex could be reliably identified in the field by experienced ringers. Our selected species are the Eurasian blackcap (“blackcap”; *Sylvia atricapilla*), barred warbler (*Sylvia nisoria*) and common redstart (“redstart”; *Phoenicurus phoenicurus*). These three species breed in Europe and Western Asia and spend the main non-breeding season in the Afrotropical zone. The blackcap captured in Israel during migration are a mixture of breeding populations spanning from eastern to northern Europe, and its main non-breeding range spans across different regions in Africa^61^ (Fig. 1). The barred warbler breed in a wide range across Europe and Asia and migrate via a narrow corridor to a rather restricted non-breeding area in East Africa (Fig. 1). The redstart captured in Israel is probably from breeding populations in eastern Europe and Siberia^62^, and spend the main non-breeding season in a wide range that includes sub-Sahara and the Arabian peninsula (Fig. 1).

The birds were caught systematically using mist- nets positioned in permanent locations throughout the study period. We considered only individuals captured during the main spring migration season (March–May), as over 96% of captured individuals were recorded on passage during these months in the study species. Each bird was aged, sexed and carefully measured by experienced ringers (wing length, fat score and weight), and date of capture was recorded. We extracted spring arrival dates in Israel as the first ordinal date of capture (days from 1st January). For each individual we only considered the first capture in a migration season. Recaptures during the same season were excluded from the analysis.

### Environmental conditions on the Afrotropical non-breeding grounds

We calculated the monthly-normalized enhanced vegetation index (EVI) during the pre-migratory period (prior to the birds’ departure; February/March) for the period 2000–2017 in the species-specific non-breeding areas based on distribution maps from BirdLife International^63^. We downloaded original spatially explicit GIS data from BirdLife website. Using qgis 3.6.0 (qgis 2018, http://qgis.osgeo.org), we selected the non-breeding range based on the parameter “season” of the attribute table of the dataset. The EVI is relevant to vegetation phenological stages^64,65^ and can be used as a proxy measure for the actual ecological conditions (e.g., food availability). The EVI was developed to determine photosynthetic activity in high biomass regions^66^. A recent study identified the main non-breeding areas of the blackcap and barred warbler from our study site using stable isotope analysis from feathers (Raz et al. in prep), and we narrowed down the range of the blackcap in accordance, to include only Ethiopia, Eritrea and eastern Sudan (Fig. 1). For the redstart, we focused on the eastern part of its vast non-breeding range (east of 15.7 degrees), as the individuals from the western part are likely migrating to Europe via the western or central flyway e.g.^67^. Although there are generally two rainfall seasons, for the northern and southern part of Africa, the distribution of seasons vary considerably across Africa, mainly south of the equator^68^. The non-breeding area of the blackcap and the redstart overlap to some extent (in Ethiopia), while the barred warbler spend the non-breeding season in southern latitude, mainly in Kenya (Fig. 1). While some differences are expected in the growing seasons between the non-breeding areas of the three species^68^, all three areas show the start of season with increasing EVI during the months prior to migration (February–March; Fig. S1).

We used Google Earth Engine (GEE), a cloud-based remote-sensing technology^69^, in order to calculate average EVI within the habitat ranges for each species. With the GEE framework, a user can upload a program code to the high-power computing architecture, which stores the large array of satellite images. Here we accessed GEE’s EVI dataset created based on 16-day composites from the daily dataset of MODIS sensor images (250 m resolution) available since February 2000. We uploaded the range maps for each species to the GEE cloud facility, and wrote a script to extract the EVI values within their extents for each time period and to calculate the average EVI within the wintering habitat range for each species. We repeated this process for all the images corresponding to the duration of time periods between 2000–2017. Then we calculated the monthly EVI average for each of the pre-migratory months February and March over the years for each species.

### Statistical analysis

#### Differences in wing length between arrival phases

We applied linear models to analyse the differences in wing length between the early and late arriving individuals. To this end, we divided our bird capture data for the entire migration season into *early* and *late* arrival phases. While arbitrary, by using 30% of each end of the arrival period we can capture a substantial proportion of early and late arrivals. Each phase comprised the first and last 30% of individuals arriving at the JBO during the migration season, respectively. We used annual mean wing length as the response variable with arrival phase (early or late), sex and age as explanatory variables, with interaction terms between arrival phase to sex and age. We accounted for age in the statistical model because some species show morphological differences in wing structure between age groups^70^. As a post-hoc test, we calculated the least square means to evaluate differences between the groups when interaction terms were significant and compared them using the *Tukey* method. For the redstart, due to low sample size (Table S1), we also ran a GLMM for the individual wing length across the study period (to avoid dividing the dataset into *early* and *late* phases and lose individual measurements). The wing length was set as a response variable of the model with arrival day, sex, age and the interactions between arrival day and sex as well as arrival day and age as the response variable, while year of capture was also set as a random effect.

#### Overall change in average wing length

We applied a general linear mixed effect model (GLMM) to describe any temporal changes in wing length over the study period, while accounting for year as a random effect. Annual mean wing length was set as a response variable with sex, age and year of capture as explanatory variables, as well as the interactions between sex and year and age and year to account for any differences in the slopes between the sexes and age groups.

#### Overall trend in spring arrival phenology

Changes in arrival phenology are dynamic and may include advanced, delayed and stable trends^71^. We therefore estimated trends in the migration phenology of each species (2000–2017) using three population quantiles: the 30th, 50th (median) and 70th percentile dates. For this purpose, we used linear quantile mixed model (lqmm)^72,73^, where separate regression lines are fitted for each of the quantile specified, thus allowing an estimation of separate trends for different phases of arrival timing of individual birds^28^. Negative and positive slopes depict that migration was advanced or delayed, respectively. The lqmm extend the quantile regression models^74^ to include random slopes.

Because in many species males migrate earlier than females^75^, we predicted inter-sexual differences in arrival timing. We therefore ran separate models for each sex in order to avoid a biased sample size (i.e. more males in the early phase). The model included ordinal day as the response variable and year, age (second year or adult) and an interaction between year and age as explanatory variables, with year also set as a random effect. We accounted for age (second year or adults) in the statistical models because some species show morphological differences in arrival timing between age groups^76,77^. Year was included as both fixed and random effect to address our question with the appropriate degrees of freedom (number of years), and taking into account yearly variations.

#### Environmental conditions

We used a GLMM with year as both fixed and random effect to first analyse the long-term trend in EVI at each of the species-specific non-breeding areas in our data. We then used GLMM to analyse the relationship between arrival dates to the stopover site in Israel and environmental condition (EVI) in both sexes in each species. To this end, we set the mean annual arrival day as the response variable with average EVI in February, average EVI in March (the months prior to migration), sex and year of capture as explanatory variables. We also included an interaction term between sex and year, and year as a random effect.

All statistical analyses were conducted using *R* version 3.6.0 (R Development Core Team)^78^. *R*-function *lme* in *R* package *nlme*^79^ was used for the mixed effect models, *R*-function *lsmeans* in the *R* package *lsmeans*^80^ was used to perform the post hoc test and calculate least square means, and the function *lqmm* in the *R* package *lqmm*^73^ was used for the linear quantile mixed model. Variables were excluded from models based on a threshold significance level set to *P* = 0.05. We confirmed the use of random effect in the relevant models by comparing the AIC of the best model with and without random effect using the REML method^81^.

## Results

### Wing length morphology in the arrival phases

**Figure 2:**
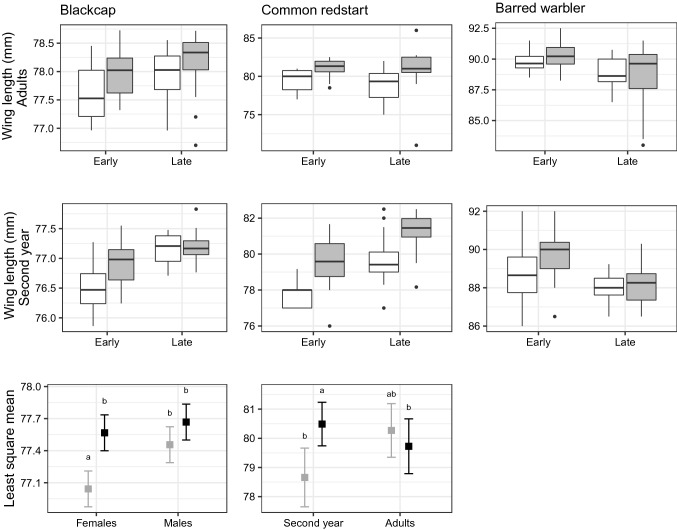
Wing length differences between arrival phases in the study species. Box-and-whisker describing the mean yearly wing length of adults (first row) and second year (second row) migratory passerines caught at a stopover site in Israel during spring migration. On the x-axis, early and late arrivals of males (grey) and females (white). Early and late arrivals were calculated as the first and last 30% of individuals on their first capture, separated for males and females. Black stripes depict the median. Statistical models are detailed in Table 1. In the third row, least square (LS) means of wing length are shown in the two arrival phases (grey- early, black-late) separately for sex (in the blackcap) and age (in the redstart). Error bars indicate the 95% confidence intervals of the LS means. Means sharing a letter are not significantly different (Tukey-adjusted comparisons). This post hoc test was done following a significant interaction between sex and group in the model for the blackcap and age and group in the model for the redstart (Table 1).

All three species showed age-dependent differences in wing length, with wings of adult birds significantly longer than second year birds. Among blackcaps the interaction between sex and group was significant (β = − 0.31, *P* = 0.02, Table 1), and a post hoc test revealed that the late arrival group had significantly longer wings than those of early arrival group only in females (β = − 0.52, t = − 5.58, *P* < 0.001, Fig. 2, Table S2). Among the barred warblers, the early arrival group had significantly longer wings than that of the late arrival group, in both second year and adult birds (β = − 1.26, *P* < 0.001, Fig. 2 and Table 1). There was no significant difference in wing length between males and females barred warbler (*P* = 0.14,). Among redstarts, the interaction between age and group was significant (β = − 2.37, *P* < 0.01, Table 1), and a post hoc test revealed that late arrivals had longer wings than early arrivals only in the second year age group (β = − 1.83, t = − 3.70, *P* = 0.008, Fig. 2, Table S2 and Table 1). In addition, the wings of males were longer than those of females in both second year and adult redstart (β = − 1.62, *P* < 0.001, Table S2). A summary of the least square means for each group is presented in Table S3. The GLMM with individual wing length measurements of the redstart show similar results. Wing length increased with arrival day, but following a significant interaction between day and age (β = − 0.03, *P* < 0.005) we ran separate models for the age classes and found that the increase was apparent only in the second year birds (Table S4, Fig. S2).

**Table 1:** Models comparing the wing length of early and late arrivals.

Blackcap	Group	Sex	Age	Group*Sex	Group*Age	F	R^2^
Group + Sex + Age + Group*Sex	0.52***	0.41***	0.98***	− 0.31*		67.66_4,139_	0.65
Group + Sex + Age + Group*Sex + Group*Age	0.61***	0.41***	1.07***	− 0.31*	**− **0.17	54.75_5,138_	0.66
**Barred warbler**							
Group + Age	− 1.26***		0.67**			17.39 _2,128_	0.20
Group + Sex + Age	− 1.25***	0.37	0.70**			12.45_3,127_	0.21
Group + Sex + Age + Group*Sex	− 0.93**	0.70*	0.69**	**− **0.67		9.87_4,126_	0.21
Group + Sex + Age + Group*Sex + Group*Age	**− **0.79	0.70*	0.82*	**− **0.69	**− **0.27	7.91_5,125_	0.21
**Common redstart**							
Group + Sex + Age + Group*Age	1.83***	1.62***	1.61**		− 2.37**	8.19 _4,100_	0.22
Group + Sex + Age + Group*Sex + Group*Age	1.86**	1.65**	1.61**	**− **0.05	− 2.38**	6.48 _5,99_	0.21

Model selection table and slopes for each variable in the linear models comparing the wing length of individuals from the early (first 30%) and late (last 30%) arrival groups (“Group”), with Sex and Age (second year or adults) as response variables. F-Statistic is presented for each model with degrees of freedom as subscripts. Post hoc tests were performed on models with significant interaction (Table S2).

**Figure 3:**
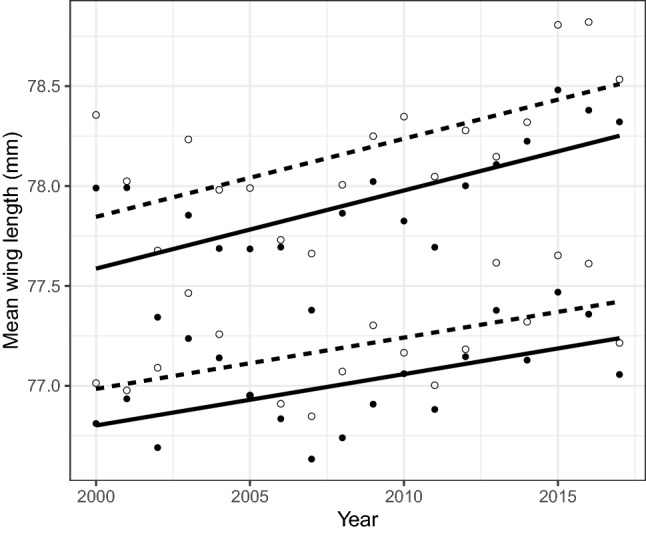
Temporal trend in the wing length of adult blackcaps (open circles denote males, solid circles denote females), captured in Israel during spring migration. Lines were fitted with a general linear mixed effect model (GLMM) with year and sex as explanatory variable, and year also as a random effect for males (dashed line) and females (solid line) in adults (upper lines) and second year birds (lower lines).

The average wing length of the blackcaps increased slightly throughout the study period (β = 0.025, *P* = 0.02, Fig. 3, Table 2) in both sexes and age groups from a general average of 77.54 mm in 2000 to 77.78 mm in 2017. A significant interaction between year of capture and age implies a different slope for second year and adult birds (Interaction term year*age: t = 2.18, *P* = 0.03), however, the difference between the slopes was minute (β = 0.01, *P* = 0.03). We did not detect any long-term trend in average wing length in the other species.

**Table 2:** Summary of model testing trend in the blackcap wing length.

Effect	Slope (β)	SE	Df	t-value	p-value
**Blackcap**					
Intercept	25.39	19.48			
Year	0.025	0.01	16	2.65	0.02
Sex	0.22	0.03	51	6.92	< 0.001
Age	− 26.03	12.36	51	− 2.10	0.04
Year*Age	0.01	0.01	51	2.18	0.03

Results of linear mixed-effect model testing the effect of Year, Age and Sex (with Year also as random effect) on the average wing length of blackcap captured during spring migration in Jerusalem, Israel during the study period (2000–2017).

### Overall change in phenology

**Figure 4:**
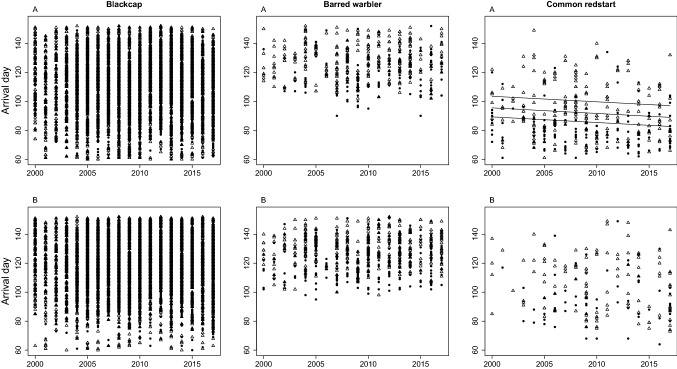
Spring migration phenology (first arrival day) of the three study species migrating from non-breeding grounds in sub-Sahara to stopover sites in Israel across 18 years (2000–2017). Data is separated for (**A**) males and (**B**) females and for adults (solid circles) and second year birds (open triangles). Lines were fitted with a linear quantile mixed model for the 30th percentile day (bottom line), median day (middle line) and 70th percentile day (upper line). Lines are shown only where slope was significant (*P* < 0.05).

In all three species, second year individuals arrived later than adults (Table 3) and the interaction term between year and age was non-significant in all models. We found that male redstart advanced arrival by 0.39 days per year throughout the study period, in all three populations measurements: 30th, median and 70th percentile dates (Table 3, Fig. 4). Female redstart, as well as both males and female blackcap and barred warbler did not show a significant change in arrival day (Table 3, Fig. 4, Table S5).

**Table 3:** Changes in arrival day across the study period and between age groups.

Percentile	Sex	Mean day		ß slope	SE	p value
**Blackcap**						
30th	M	103	*Year*	< 0.001	< 0.001	0.67
Median	M	111		0.01	0.007	0.45
70th	M	119		0.01	0.006	0.38
30th	M		***Age***	**− 0.41**	**0.05**	** < 0.001**
Median	M			**− 0.35**	**0.04**	** < 0.001**
70th	M			**− 0.35**	**0.05**	** < 0.001**
30th	F	111	*Year*	0.01	0.01	0.25
Median	F	119		0.00	0.01	0.60
70th	F	127		0.00	0.01	0.65
30th	F		***Age***	**− 0.19**	**0.03**	** < 0.001**
Median	F			**− 0.31**	**0.05**	** < 0.001**
70th	F			**− 0.30**	**0.06**	** < 0.001**
**Barred warbler**						
30th	M	119	*Year*	0.19	0.024	0.43
Median	M	126		0.19	0.024	0.42
70th	M	133		0.19	0.024	0.42
30th	M		***Age***	**− 9.85**	**1.28**	** < 0.001**
Median	M			**− 8.99**	**1.57**	** < 0.001**
70th	M			**− 7.34**	**1.28**	** < 0.001**
30th	F	121	*Year*	0.15	0.13	0.27
Median	F	127		0.15	0.13	0.26
70th	F	135		0.15	0.13	0.25
30th	F		***Age***	**− 7.81**	**0.91**	** < 0.001**
Median	F			**− 6.89**	**1.04**	** < 0.001**
70th	F			**− 5.80**	**0.94**	** < 0.001**
**Common redstart**						
30th	M	77	***Year***	**− 0.39**	**0.14**	**0.01**
Median	M	85		**− 0.39**	**0.14**	**0.01**
70th	M	95		**− 0.38**	**0.14**	**0.01**
30th	M		***Age***	**− 14.36**	**1.73**	** < 0.001**
Median	M			**− 14.36**	**1.73**	** < 0.001**
70th	M			**− 14.36**	**1.73**	** < 0.001**
30th	F	88	*Year*	**− **0.51	0.37	0.17
Median	F	99		**− **0.51	0.37	0.17
70th	F	114		**− **0.50	0.37	0.18
30th	F		***Age***	**− 7.61**	**3.31**	**0.03**
Median	F			**− 10.05**	**2.76**	** < 0.001**
70th	F			**− 13.54**	**3.13**	** < 0.001**

The changes in first arrival day (days per year) of the three studied species to stopover site in Israel, estimated by linear quantile mixed models (first 30th percentile day, median and 70th percentile day) and the differences between age groups (adults and second year. The slope for age is relative to second year birds, i.e. negative slopes denote earlier arrival of adult birds. Mean arrival day (“Mean day”) is calculated for adults and second year birds combined. Significant results (p < 0.05) are in bold. Significant advancement of arrival is found only for male redstart. SE: standard error.

### Linking arrival timing to environmental conditions

**Figure 5:**
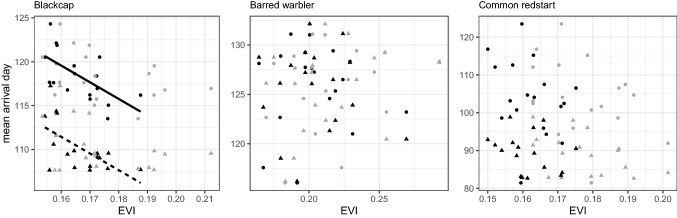
The relationship between mean arrival day and average Enhanced Vegetation Index (EVI). EVI is calculated at the species-specific non-breeding grounds during the years 2000–2017 in the months prior to migration departure: February (black symbols) and March (grey symbols) in the three study species. Triangles denote males and circles denote females. Predicted lines for males (dashed line) and females (solid line) are drawn from a general linear mixed effect model (GLMM) with year as both fixed and random effect for significant slopes in the blackcap (*P* < 0.05).

EVI in February at the non-breeding area of the blackcap significantly decreased over 18 years (β = − 0.001, t = -2.67, *P* = 0.02) while there was no significant change in EVI in March (*P* = 0.25). In the non-breeding area of the redstart, EVI significantly increased in both February and March (β < 0.001, t = 2.84, *P* = 0.01 and β = 0.001, t = 3.11, *P* < 0.01, respectively) and in the non-breeding area of the barred warbler we did not detect any trend in EVI in both February (*P* = 0.42) and March (*P* = 0.80). We found a significant negative effect of EVI in February at the African non-breeding grounds on the mean arrival day of female and male blackcap (t = − 3.38_14_, *P* < 0.01), i.e. increased EVI values correlated with advanced arrival of the blackcap. We did not find any significant effects of EVI on arrival dates of the barred warbler or the redstart (Table 4, Fig. 5).

**Table 4:** Summary of models testing the effect of EVI on arrival timing.

**Blackcap**	AIC	EVI February	EVI March	Sex	Year	Sex:Year	df
EVI_feb_ + EVI_mar_ + Sex + Year	136.54	− 215.30**	**− **20.02	− 8.12***	**− **0.19		7
EVI_feb_ + EVI_mar_ + Sex + Year + Year:Sex	141.86	− 215.30**	**− **20.02	23.58	**− **0.18	**− **0.01	8
EVI_feb_ + Sex	142.69	− 189.66***		− 8.12***			5
EVI_feb_ + Sex + Year	143.74	− 233.63***		− 8.12***	**− **0.19		6
**Barred warbler**							
EVI_feb_ + EVI_mar_ + Sex + Year + Year:Sex	188.67	**− **44.37	52.85	610.86	0.18	**− **0.30	8
EVI_feb_ + EVI_mar_ + Sex + Year	188.36	**− **44.37	52.85	**− **0.40	**− **0.03		7
EVI_feb_ + EVI_feb_ + Sex	184.95	**− **47.11	54.47	**− **0.40			6
EVI_mar_ + Sex	193.50		32.81	**− **0.40			5
EVI_mar_	193.27		32.81				4
**Common redstart**							
EVI_feb_ + EVI_mar_ + Sex + Year	224.91	**− **70.30	**− **184.26	− 14.98***	− 0.52*		7
EVI_feb_ + EVI_mar_ + Sex + Year + Year:Sex	226.09	**− **44.09	**− **195.86	**− **676.32	**− **0.68	0.33	8
EVI_mar_ + Sex + Year	235.56		**− **206.64	− 14.89***	− 0.53*		6

Model selection and slopes for each variable in a general linear mixed effect model (GLMM) to describe the relationship between mean arrival day and Enhanced Vegetation Index (EVI) at the species-specific non-breeding grounds during the years 2000–2017 in the months prior to migration departure (February and March) in the three study species. Best models appear in the first line. We started with the most complicated models (i.e. including all variables) and excluded variables based on significance level set to *P* = 0.05. Significance code: ^**.**^ 0.06, * < 0.05, ** < 0.01, *** < 0.001.

## Discussion

In this study, using a combination of long-term biological observations and satellite-based environmental monitoring, we show inter-sexual differences in changes in arrival timing, with the arrival timing of male redstart advancing in recent decades, independently from the environmental conditions during the pre-migratory period. On the other hand, the arrival day of the blackcap was significantly linked to EVI on its non-breeding ground, while no consistent trend in arrival phenology was detected. In line with our expectations, little variation in environmental conditions at the non-breeding site of the barred warbler were matched with unchanged arrival timing. We also confirmed morphological differences in wing length in the barred warbler, with individuals arriving later having shorter wings, which suggests multiple populations, most likely at the breeding ground.

### Wing length as a proxy for population structure and behaviour

In the barred warbler, individuals arriving later had significantly shorter wings than early arrivals in both sexes and ages. The longer wings suggest that the early passage may head to breeding sites further than the later arrivals. The possibility that they arrive from southern locations of non-breeding areas is less likely given the limited range of the barred warbler in Africa (Fig. 1). Given the suggested geographical segregation between populations, we would expect that if phenological changes occur in this species, they could vary between populations (measured as arrival percentiles in this study), depending on the cues they are responding to along their migration route and breeding/non-breeding condition. In the redstart, early arrivals had shorter wings, but the difference was significant only among the second year birds. It could be therefore related to wing development rather than actual difference between populations. In the blackcap, early females had shorter wings than late females. This trend is partially in agreement with a previous study on the blackcap in Israel^61^, which suggested that blackcaps (both sexes) from the early migration phase had shorter wings than the later arrivals. The same study found that the shorter wing individuals breed at a more southerly or westerly destination in Europe^61^. The longer wings of the later arrivals may therefore facilitate adjustment to the fast spring vegetal growth in breeding grounds in eastern Europe compared to the western range^82^. However, the difference in wing length between the arrival groups was very small (< 1 mm), and could be attributed to measurements error. Interpretation of these differences should be therefore done with caution.

The average wing length of the blackcap increased throughout the study period (Fig. 3 and Table 1) in agreement with Kovács, et al.^54^, suggesting that changes had taken place in the population structure and/or in migration behaviour. Such a change in the morphology of a species may result from selection pressure for individuals with longer wings, because long wings are more energetically efficient for long flights and may facilitate the adjustment to changing conditions along the migration route^56,82^. Secondly, the blackcap’s high adaptability^45,83^, may also have allowed a shift in the migration route that altered the ratio of long/short wings in individuals passing over Israel. Although significant, we must be careful with interpreting this change in wing length, as the increase in wing length was very small (< 1 mm), and therefore errors in measurements cannot be excluded as an explanation.

### Overall changes in migration phenology and links to environmental conditions at the non-breeding grounds

#### Common redstart

We show that male redstart advanced arrival to stopover site throughout the study period, but that female redstart did not show a similar trend. Although previous studies found that individuals arriving at the early phase advanced arrival timing more than individuals from later arrival phases e.g.^3,71,84^, redstart arriving to Israel from sub-Sahara show advanced arrival across all arrival timing percentiles. This result is further supported by the similar wing length of adults across arrival phases, suggesting that the redstart arriving to Israel may originate from the same population, although it is also possible that the multiple populations simply do not differ in wing length. Our results partially agree with previous studies. Tøttrup and Thorup^60^ and Tryjanowski et al.^85^, found that both male and female redstarts advanced their arrival timing at a stopover site in the Baltic Sea between 1976–1997, and Maggini, et al.^7^ showed advanced arrival of both males and females to a stopover site in Italy between 2002–2019. In addition, Newson, et al.^86^ present earlier arrival of redstart to breeding grounds in the UK between 2002–2011 than in the mid-1960s, and Porkert et al.^62^ demonstrated advanced egg-laying dates in nine Eurasian populations of the redstart. The advanced arrival of both sexes at the breeding ground, in contrast to the trend found only in males in Israel, may be a result of females adjusting migration speed *en route*^5,67,87^. Sexual differences in changes to migration timing were also shown in willow warbler *Phylloscopus trochilus* in Sweden, where males advanced arrival more strongly than females^84^. These sexual differences may be a result of climate change causing greatest advancement in species with stronger female selection^88^, or may be attributed to additional forces that act on female phenology such as timing of food resource availability. Yosef and Wineman^89^, for example, suggested that female blackcap place more importance on energy-maximization, while males use a time-minimization strategy.

In agreement with previous studies^67,90^, we could not link between local vegetation phenology and departure timing of the redstart from the African non-breeding grounds. Given the large range of redstart on the African non-breeding ground, it is likely that we failed to detect such a trend due to the insufficient range accuracy for the populations flying through Israel. There is therefore a need to focus on identifying, at the population level, the African non-breeding areas where data is very limited, to allow accurate evaluation of the spatial and temporal resources available during the critical period of pre-migratory fattening. Secondly, the departure timing of the redstart may be independent of seasonal change in vegetation. Redstart forage on insects on the ground^91^ which are less dependent on green-leaves, and the EVI may not represent true food availability. The advancement in arrival timing of the redstart may therefore be a result of climate-driven evolutionary change^3^, or more affected by other climatic variable such as rain and tail wind^16^, explanations which are beyond the scope of this study.

#### Blackcap

Similarly to Tøttrup and Thorup^60^, and Tryjanowski et al.^92^ that found no evidence of changing arrival trend in the blackcap at a stopover site in Europe until the late 1990s, the blackcap migrating through Israel during our study period did not show any change in arrival timing. Also Maggini, et al.^7^ did not find a trend in arrival timing of the blackcap departing from North Africa to stop over site in Italy between 2002–2019. In contrast, Ożarowska, et al.^93^ found a weak, but significant advancement of the blackcap arrival at another ringing station at the Baltic Sea during spring migration in 1994–2009.

While migratory restlessness in the blackcap was shown to be directly affected by genetic control^24^, the decision when to depart combines endogenous and exogenous cues. We found that high values of EVI at the African non-breeding grounds correlated to advanced arrival of blackcap at the stopover site (i.e. negative slope). This relationship is in agreement with Jonzén et al.^3^, which found that low productivity (measured by high NAO index) delayed spring arrival of migrants from non-breeding area south of the Sahara Desert to a stopover site in Italy. In contrast, Tøttrup et al.^5^ suggested that high values of NDVI allowed migratory blackcaps to benefit from improved conditions on the African non-breeding grounds before crossing the Sahara Desert, thus delaying their departure. These discrepancies may be a result of both the different indices used (NDVI vs EVI), and the area from which the index was calculated. Tøttrup, et al.^5^ based their non-breeding area calculation on species distribution models^94^, encompassing a wider area. Our assumed non-breeding area was calculated based on data from BirdLife International^63^, and confirmed and further restricted by stable isotope analysis from feathers of blackcaps collected at our study site, during spring migration in the years 2017–2018 (Raz et al. in prep).

#### Barred warbler

In agreement with previous studies in Poland^85^ and in Eilat, Israel^95^, we did not detect any change in the arrival timing of the barred warbler at the stopover site during spring migration. The arrival day of barred warbler was also independent of spatial and temporal availability of resources during the pre-migratory period (measured as EVI). Combined with the relatively stable environmental conditions on its non-breeding ground during the study period, the decision when to depart on migration is likely more dominated by endogenous cues, photoperiod^22,33,67^ or environmental variables other than vegetation growth^16^. While internal cues are fundamentals of the annual cycle in the evolution of migratory birds^24^, in the case of low phenotypic plasticity, it limits the ability of individuals to respond to climate change and risk its fitness and survival e.g.^9^. Indeed, sub-optimal fattening resources as a result of dry conditions on the non-breeding staging grounds in eastern Africa were linked to low survival of the barred warbler and its extinction from the eastern Baltic in the late 1980’^36^. High flexibility in migration timing and adjustability to the local conditions could possibly improve the response of the species to changes, and increase its survival.

## Conclusions

Our study species show flexible departure and arrival timings to stopover sites in some populations (the blackcap and redstart), and confirms an advanced trend in male redstart, but not in the other species. We also suggest low plasticity in the barred warbler, which may be dominated primarily by external and internal cues independent of the actual state of the biotic environment. While increasing global warming in general resulted in advancement of spring activities^96^, still some long distance migrants departing from sub-Sahara regions, where long-term global changes occur at a slower pace^1,2^ and extreme events are common^97^, are arriving at warmer breeding grounds and are accumulating “thermal delay”^9^. Given particularly rapid rates of change in environmental conditions at higher latitudes, these migrants may experience difficulty in the race to match global changes to ensure their survival.

## Supplementary Information

Supplementary Information 1.

Supplementary Information 2.

Supplementary Information 3.
