# Supplementary material for: Limited flexibility in departure timing of migratory passerines at the East-Mediterranean flyway
Source: Sci Rep. 2021 Mar 4;11:5184. doi: 10.1038/s41598-021-83793-x (PMC7933344; doi:10.1038/s41598-021-83793-x)
Supplement: Supplementary file 1 — Supplementary Information 1. [file 41598_2021_83793_MOESM1_ESM.docx]

**-Supplementary Information-**

**Limited flexibility in departure timing of migratory passerines at the East-Mediterranean flyway**

Yaara Aharon-Rotman, Gidon Perlman, Yosef Kiat, Tal Raz, Amir Balaban and Takuya Iwamura

**Table S1**. Summary table of sample size (number of individuals) in each group of the three study species used in the analysis. SY= Second year birds.

|  | **Blackcap** | | | | **Barred** | | | | **Redstart** | | | |
| --- | --- | --- | --- | --- | --- | --- | --- | --- | --- | --- | --- | --- |
| **Year** | Females | Males | SY | Adults | Females | Males | SY | Adults | Females | Males | SY | Adults |
| 2000 | 474 | 276 | 463 | 166 | 15 | 8 | 15 | 9 | 11 | 16 | 10 | 10 |
| 2001 | 373 | 285 | 374 | 210 | 18 | 11 | 27 | 5 | 4 | 16 | 7 | 9 |
| 2002 | 511 | 437 | 528 | 349 | 26 | 14 | 24 | 15 | 6 | 6 | 6 | 1 |
| 2003 | 880 | 1045 | 981 | 831 | 23 | 14 | 27 | 12 | 11 | 14 | 15 | 5 |
| 2004 | 929 | 941 | 856 | 932 | 72 | 34 | 65 | 47 | 11 | 25 | 20 | 10 |
| 2005 | 1030 | 995 | 1090 | 791 | 70 | 22 | 66 | 30 | 15 | 28 | 24 | 18 |
| 2006 | 1022 | 937 | 850 | 997 | 12 | 16 | 15 | 14 | 15 | 27 | 25 | 16 |
| 2007 | 834 | 791 | 680 | 821 | 47 | 34 | 53 | 28 | 13 | 22 | 17 | 13 |
| 2008 | 1143 | 1101 | 1257 | 711 | 58 | 47 | 65 | 43 | 18 | 32 | 28 | 15 |
| 2009 | 1134 | 1087 | 1133 | 782 | 51 | 50 | 55 | 48 | 29 | 30 | 34 | 22 |
| 2010 | 1192 | 1022 | 1164 | 925 | 80 | 34 | 73 | 43 | 14 | 23 | 22 | 13 |
| 2011 | 1134 | 950 | 1024 | 928 | 47 | 28 | 47 | 35 | 8 | 19 | 20 | 6 |
| 2012 | 1275 | 1087 | 1312 | 957 | 50 | 24 | 47 | 30 | 12 | 16 | 10 | 13 |
| 2013 | 1166 | 1114 | 1349 | 895 | 105 | 54 | 118 | 60 | 16 | 20 | 18 | 16 |
| 2014 | 1310 | 1177 | 1305 | 1093 | 62 | 51 | 75 | 41 | 16 | 26 | 19 | 24 |
| 2015 | 1012 | 977 | 909 | 973 | 25 | 19 | 21 | 24 | 9 | 16 | 15 | 7 |
| 2016 | 881 | 822 | 862 | 801 | 57 | 29 | 33 | 61 | 11 | 18 | 19 | 8 |
| 2017 | 1110 | 903 | 1109 | 880 | 37 | 21 | 37 | 25 | 24 | 26 | 24 | 27 |

**Table S2.** Results of post-hoc estimated marginal means test for multiple comparisons with Tukey method for adjusted p-value to test differences in wing length between early and late arrivals. Post-hoc test was performed following a significant interaction between group and sex in blackcap and group and age in redstart. SY= Second year birds.

| **Blackcap** | **Estimate** | **SE** | **df** | **t-ratio** | **p-value** |
| --- | --- | --- | --- | --- | --- |
| **Contrast** |  |  |  |  |  |
| **Early F- late F** | **-0.52** | **0.09** | **139** | **-5.58** | **<0.001** |
| Early M -late M | -0.21 | 0.09 | 139 | -2.26 | 0.11 |
| **Early F- early M** | **-0.41** | **0.09** | **139** | **-4.39** | **<0.001** |
| Late F- late M | 0.10 | 0.09 | 139 | -1.07 | 0.71 |
| **Common redstart- Adults** |  |  |  |  |  |
| Early F- late F | 0.54 | 0.52 | 100 | 1.03 | 0.97 |
| Early M -late M | 0.54 | 0.52 | 100 | 1.03 | 0.97 |
| **Early F-early M** | **-1.62** | **0.36** | **100** | **-4.44** | **<0.001** |
| **Late F-late M** | **-1.62** | **0.36** | **100** | **-4.44** | **<0.001** |
| **Common redstart- SY** |  |  |  |  |  |
| **Early F- late F** | **-1.83** | **0.49** | **100** | **-3.70** | **0.008** |
| **Early M -late M** | **-1.83** | **0.49** | **100** | **-3.70** | **0.008** |
| **Early F- early M** | **-1.62** | **0.36** | **100** | **-4.44** | **<0.001** |
| **Late F- late M** | **-1.62** | **0.36** | **100** | **-4.44** | **<0.001** |

**Table S3.** Summary table of least square means (“Mean”) for wing length and its lower and upper confidence intervals for each group (arrival phase (early or late), age and sex) in the three study species. SY= Second year birds.

| **Blackcap**  **Blackcap** | | **Age** | **Sex** | **Mean** | **SE** | **df** | **lower** | | **upper** | |  |
| --- | --- | --- | --- | --- | --- | --- | --- | --- | --- | --- | --- |
| Early | | SY | F | 76.55 | 0.07432 | 139 | 76.4 | | 76.7 | |  |
|  | |  | M | 76.96 | 0.07432 | 139 | 76.82 | | 77.11 | |  |
|  | | Adults | F | 77.53 | 0.07432 | 139 | 77.39 | | 77.68 | |  |
|  | |  | M | 77.95 | 0.07432 | 139 | 77.8 | | 78.09 | |  |
| Late | | SY | F | 77.07 | 0.07432 | 139 | 76.93 | | 77.22 | |  |
|  | |  | M | 77.18 | 0.07432 | 139 | 77.03 | | 77.32 | |  |
|  | | Adults | F | 78.06 | 0.07432 | 139 | 77.91 | | 78.21 | |  |
|  | |  | M | 78.16 | 0.07432 | 139 | 78.01 | | 78.31 | |  |
| **Barred warbler** |  | | |  |  |  |  |  | |  | |
| Early | | SY | F | 88.9 | 0.275 | 126 | 88.4 | | 89.5 | |  |
|  | |  | M | 89.6 | 0.273 | 126 | 89.1 | | 90.2 | |  |
|  | | Adults | F | 89.6 | 0.268 | 126 | 89.1 | | 90.2 | |  |
|  | |  | M | 90.3 | 0.277 | 126 | 89.8 | | 90.9 | |  |
| Late | | SY | F | 88 | 0.272 | 126 | 87.5 | | 88.5 | |  |
|  | |  | M | 88 | 0.276 | 126 | 87.5 | | 88.6 | |  |
|  | | Adults | F | 88.7 | 0.272 | 126 | 88.2 | | 89.2 | |  |
|  | |  | M | 88.7 | 0.297 | 126 | 88.1 | | 89.3 | |  |
| **Redstart** |  | | |  |  |  |  |  | |  | |
| Early | | SY | F | 77.8 | 0.476 | 100 | 76.9 | | 78.8 | |  |
|  | |  | M | 79.5 | 0.395 | 100 | 78.7 | | 80.3 | |  |
|  | | Adults | F | 79.5 | 0.441 | 100 | 78.6 | | 80.3 | |  |
|  | |  | M | 81.1 | 0.368 | 100 | 80.4 | | 81.8 | |  |
| Late | | SY | F | 79.7 | 0.347 | 100 | 79 | | 80.4 | |  |
|  | |  | M | 81.3 | 0.347 | 100 | 80.6 | | 82 | |  |
|  | | Adults | F | 78.9 | 0.395 | 100 | 78.1 | | 79.7 | |  |
|  | |  | M | 80.5 | 0.43 | 100 | 79.7 | | 81.4 | |  |

**Table S4.** Linear mixed effect model selection, testing the change in wing length of redstart with arrival day (“Day”). The models include Sex and Year as explanatory variable, with the interaction between Day and Sex, and Year also as a random effect. The models were ran separately for adults and second year birds.

|  | **Adults** | | | | **Second Year** | | | |
| --- | --- | --- | --- | --- | --- | --- | --- | --- |
| **Model** | day | sex | Year | Day:Sex | day | sex | Year | Day:Sex |
| Day + Sex |  |  |  |  | **0.03** | **2.18** |  |  |
| Day + Sex + Year | <0.001 | **2.32** | **0.09** |  | **0.03** | **2.22** | 0.04 |  |
| Day + Sex + Year + Day:Sex | -0.01 | 0.51 | **0.09** | 0.02 | **0.03** | 1.42 | 0.03 | 0.02 |

**Table S5**. Summary table of median arrival day (since 1^st^ January) in each group in the three study species. SY= Second year birds

|  | **Blackcap** | | | | **Barred warbler** | | | | **Common redstart** | | | |
| --- | --- | --- | --- | --- | --- | --- | --- | --- | --- | --- | --- | --- |
| **Year** | Males | Females | SY | Adults | Males | Females | SY | Adults | Males | Females | SY | Adults |
| 2000 | 119 | 126 | 123 | 120 | 121 | 123 | 126 | 121 | 89 | 116 | 112 | 86 |
| 2001 | 112 | 124 | 118 | 117 | 123 | 122 | 123 | 121 | 74 | 123 | 95 | 75 |
| 2002 | 107 | 119 | 115 | 110 | 130 | 131.5 | 131 | 130 | 100 | 100.5 | 100 | 95 |
| 2003 | 110 | 115 | 112 | 110 | 123.5 | 127 | 127 | 118 | 94.5 | 93 | 93 | 96 |
| 2004 | 110 | 114 | 112 | 110 | 134 | 127.5 | 136 | 115 | 83 | 109 | 88 | 80 |
| 2005 | 108 | 122 | 117 | 110 | 135 | 135 | 136 | 131 | 82 | 96 | 103 | 80.5 |
| 2006 | 114 | 116 | 116 | 114 | 119 | 118 | 119 | 118 | 86 | 89 | 89 | 86 |
| 2007 | 106 | 115 | 112 | 106 | 121 | 125 | 127 | 110.5 | 89 | 105 | 103.5 | 84 |
| 2008 | 112 | 121 | 117 | 113 | 129 | 129 | 130 | 124 | 95 | 117.5 | 114 | 87 |
| 2009 | 112 | 116 | 115 | 112 | 111.5 | 115 | 115 | 113.5 | 81 | 100 | 96.5 | 78.5 |
| 2010 | 112 | 118 | 116.5 | 113 | 130.5 | 129 | 131 | 124 | 77 | 84.5 | 82.5 | 75 |
| 2011 | 113.5 | 123 | 121 | 117 | 126.5 | 131 | 134.5 | 124 | 90 | 107.5 | 100 | 78 |
| 2012 | 113 | 120 | 119 | 113 | 127 | 124 | 130.5 | 120 | 86.5 | 111 | 111 | 87 |
| 2013 | 116 | 124 | 122 | 114 | 127 | 128 | 130 | 124 | 73 | 105.5 | 96 | 75.5 |
| 2014 | 111 | 120 | 118 | 111 | 126 | 125.5 | 129 | 124 | 77 | 108 | 99 | 77 |
| 2015 | 112 | 115.5 | 115 | 112 | 120 | 133 | 133 | 114 | 79.5 | 80 | 79 | 80 |
| 2016 | 108 | 118 | 114 | 109 | 124 | 126 | 125.5 | 122 | 89 | 111 | 103 | 86.5 |
| 2017 | 110 | 121 | 121 | 110 | 127 | 128 | 134 | 124 | 80 | 89.5 | 87.5 | 81 |

**Figure legends**

**Figure S1.** Example of yearly variation in Enhanced Vegetation Index (EVI) in 2015 at the species-specific African non-breeding range (See Fig. 1 for maps of the non-breeding ranges) for the three study species. EVI was calculated over a 16-day composite from the daily remotely-sensed dataset of MODIS sensor images (250 m resolution) over the period 2000-2017 using Google Earth Engine (See methods for details).

**Figure S2.** The relationship between the individual wing length of redstart and arrival day. Arrival days (since 1^st^ January) were recorded during spring migration at the stopover site in Israel, separately for second year and adult birds. The plot show partial residuals from a linear mixed model (GLMM) with Arrival day and Sex as predictor and Year as a random effect. Grey circles and trend line denote males and black symbols denote females.
