# Supplementary figures and images for: Limited flexibility in departure timing of migratory passerines at the East-Mediterranean flyway
Source: Sci Rep. 2021 Mar 4;11:5184. doi: 10.1038/s41598-021-83793-x (PMC7933344; doi:10.1038/s41598-021-83793-x)

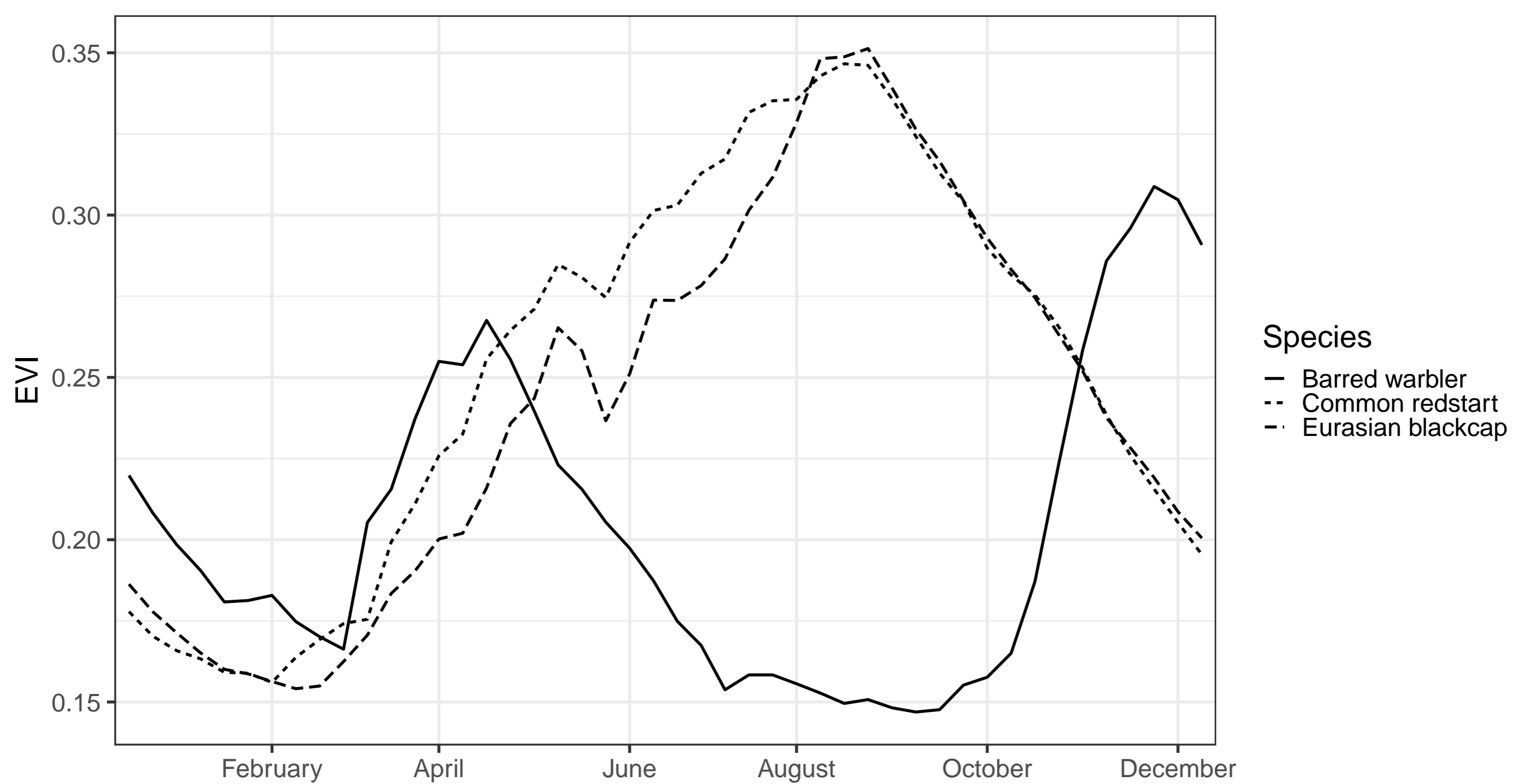

Supplement: Supplementary file 2 — Supplementary Information 2. [file 41598_2021_83793_MOESM2_ESM.pdf]

**Second year**

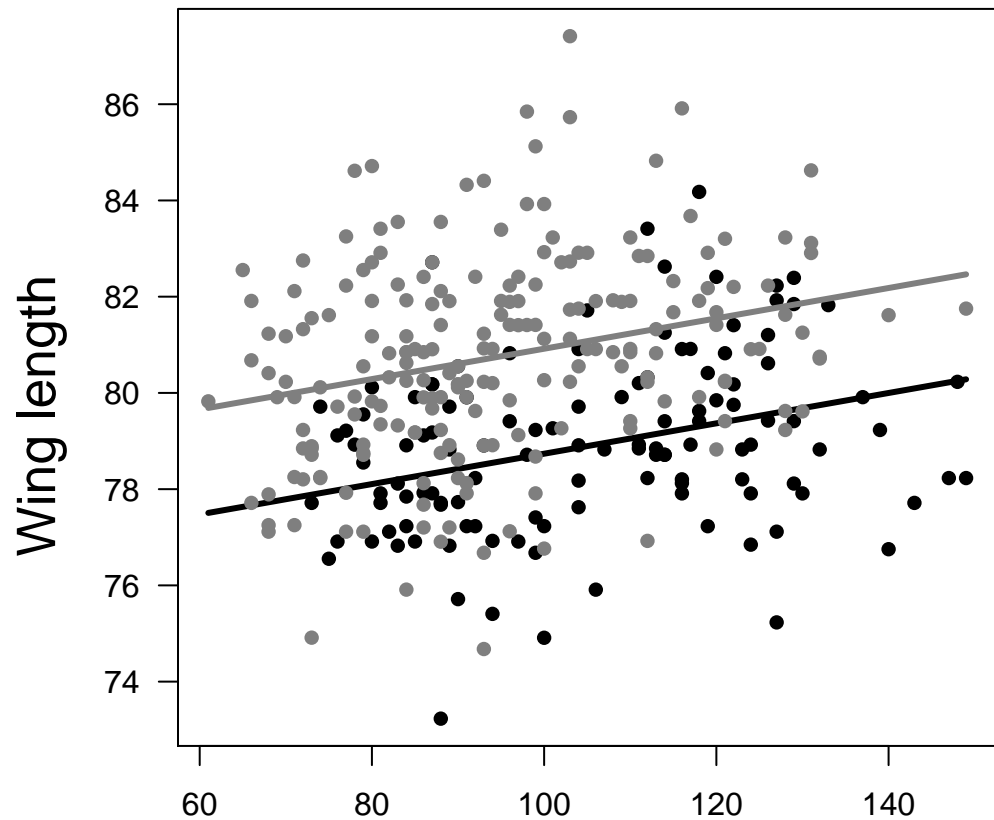

**Adults**

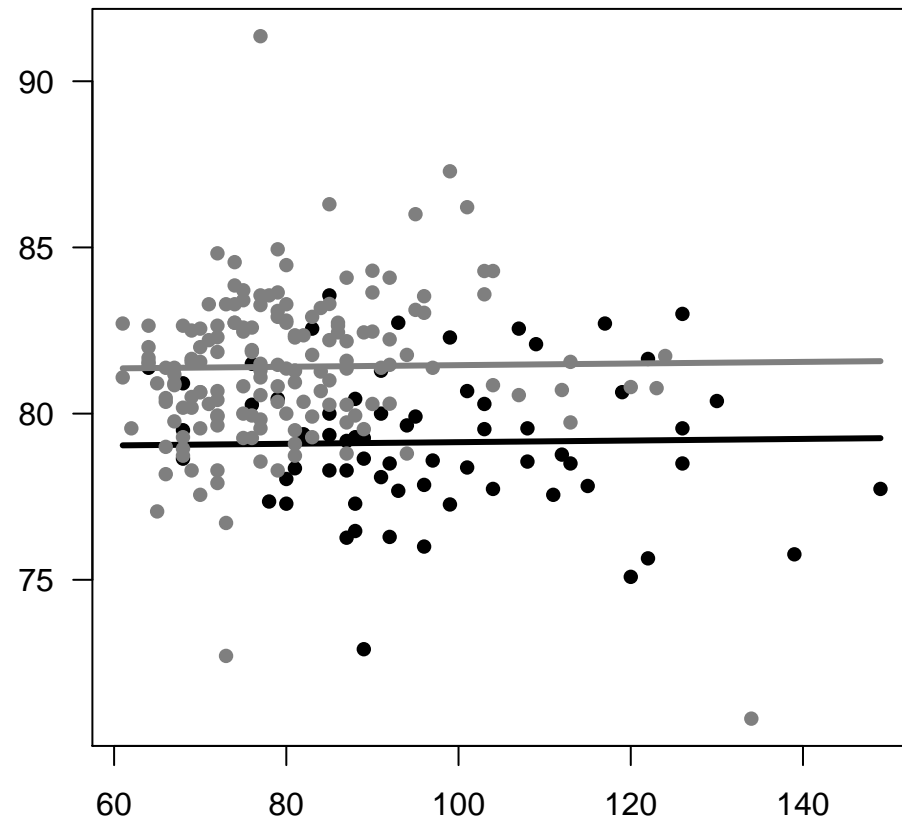

Arrival day

Supplement: Supplementary file 3 — Supplementary Information 3. [file 41598_2021_83793_MOESM3_ESM.pdf]
